# Leveraging real-world data to predict cancer cachexia stage, quality of life, and survival in a racially and ethnically diverse multi-institutional cohort of treatment-naïve patients with pancreatic ductal adenocarcinoma

**DOI:** 10.3389/fonc.2024.1362244

**Published:** 2024-07-23

**Authors:** Jennifer B. Permuth, Margaret A. Park, Dung-Tsa Chen, Toni Basinski, Benjamin D. Powers, Clement K. Gwede, Kaleena B. Dezsi, Maria Gomez, Shraddha L. Vyas, Tiago Biachi, Elena M. Cortizas, Sylvia Crowder, Maria Genilo-Delgado, B.Lee Green, Anna Greene, Christopher Gregg, Sarah E. Hoffe, Kun Jiang, Bora Kim, Vanitha Vasudevan, Jeronimo Garcialopez De Llano, Anjana A. Menon, Qianxing Mo, Lina M. MorenoUrazan, Shaffer Mok, Nathan Parker, Sahana Rajasekhara, Ghulam Rasool, Andrew Sinnamon, Lauren Sparks, Paul A. Stewart, Kenneth Tardif, Alexandra F. Tassielli, Jamie K. Teer, Dan Viet Tran, Kea L. Turner, Susan T. Vadaparampil, Christopher J. Whelan, Wade G. Douglas, Vic Velanovich, Andreas Karachristos, Adrian Legaspi, Kenneth Meredith, Manual A. Molina-Vega, Kevin L. Huguet, Juan P. Arnoletti, Mark Bloomston, Jose Trevino, Nipun B. Merchant, Jose M. Pimiento, Pamela J. Hodul, Mokenge Malafa, Jason Fleming, Sarah M. Judge, Daniel K. Jeong, Andrew Judge

**Affiliations:** ^1^ Department of Cancer Epidemiology, Moffitt Cancer Center, Tampa, FL, United States; ^2^ Department of Gastrointestinal Oncology, Moffitt Cancer Center, Tampa, FL, United States; ^3^ Department of Biostatistics and Bioinformatics, Moffitt Cancer Center, Tampa, FL, United States; ^4^ Department of Surgery, School of Medicine, University of Maryland, Baltimore, MD, United States; ^5^ Department of Health Outcomes and Behavior, Moffitt Cancer Center, Tampa, FL, United States; ^6^ Research Analytics and Development Cores, Baylor Scott & White Research Institute (BSWRI), Dallas, TX, United States; ^7^ Leonard M. Miller School of Medicine, University of Miami, Miami, FL, United States; ^8^ Department of Neurobiology, School of Medicine, University of Utah Health, Salt Lake City, UT, United States; ^9^ Department of Pathology, Moffitt Cancer Center, Tampa, FL, United States; ^10^ Center for Advanced Surgical Oncology, Palmetto General Hospital, Hialeah, FL, United States; ^11^ Supportive Care Medicine, Moffitt Cancer Center, Tampa, FL, United States; ^12^ Department of Machine Learning, Moffitt Cancer Center, Tampa, FL, United States; ^13^ Translational Research Institute, AdventHealth, Orlando, FL, United States; ^14^ Department of Surgery, St. Anthony’s Hospital, St. Petersburg, FL, United States; ^15^ Community Outreach, Engagement & Equity, Moffitt Cancer Center, Tampa, FL, United States; ^16^ Department of Metabolism and Cancer Physiology, Moffitt Cancer Center, Tampa, FL, United States; ^17^ Department of Clinical Sciences, College of Medicine, Florida State University, Tallahassee, FL, United States; ^18^ Tampa General Hospital, Tampa, FL, United States; ^19^ Department of Gastrointestinal Surgical Oncology, Sarasota Memorial Hospital, Sarasota, FL, United States; ^20^ Department of Surgical Oncology, Lakeland Regional Hospital, Lakeland, FL, United States; ^21^ Department of Surgical Oncology, Orlando Regional Medical Center, Orlando, FL, United States; ^22^ Department of Surgical Oncology, Lee Health Regional Cancer Center, Fort Myers, FL, United States; ^23^ Department of Surgery, College of Medicine, University of Florida, Gainesville, FL, United States; ^24^ Massey Cancer Center, Virginia Commonwealth University, Richmond, VA, United States; ^25^ Division of Surgical Oncology, DeWitt Daughtry Family Department of Surgery, Leonard M. Miller School of Medicine, University of Miami, Miami, FL, United States; ^26^ Department of Physical Therapy, College of Public Health and Health Professions, University of Florida, Gainesville, FL, United States

**Keywords:** cancer-associated cachexia, pancreatic adenocarcinoma, longitudinal prospective cohort, health-related quality of life, racial and ethnic disparities

## Abstract

**Introduction:**

Cancer-associated cachexia (CC) is a progressive syndrome characterized by unintentional weight loss, muscle atrophy, fatigue, and poor outcomes that affects most patients with pancreatic ductal adenocarcinoma (PDAC). The ability to identify and classify CC stage along its continuum early in the disease process is challenging but critical for management.

**Objectives:**

The main objective of this study was to determine the prevalence of CC stage overall and by sex and race and ethnicity among treatment-naïve PDAC cases using clinical, nutritional, and functional criteria. Secondary objectives included identifying the prevalence and predictors of higher symptom burden, supportive care needs, and quality of life (QoL), and examining their influence on overall survival (OS).

**Materials and methods:**

A population-based multi-institutional prospective cohort study of patients with PDAC was conducted between 2018 and 2021 by the Florida Pancreas Collaborative. Leveraging patient-reported data and laboratory values, participants were classified at baseline into four stages [non-cachexia (NCa), pre-cachexia (PCa), cachexia (Ca), and refractory cachexia (RCa)]. Multivariate regression, Kaplan Meier analyses, and Cox regression were conducted to evaluate associations.

**Results:**

CC stage was estimated for 309 PDAC cases (156 females, 153 males). The overall prevalence of NCa, PCa, Ca, and RCa was 12.9%, 24.6%, 54.1%, and 8.4%, respectively. CC prevalence across all CC stages was highest for males and racial and ethnic minorities. Criteria differentiated NCa cases from other groups, but did not distinguish PCa from Ca. The most frequently reported symptoms included weight loss, fatigue, pain, anxiety, and depression, with pain significantly worsening over time. The greatest supportive care needs included emotional and physical domains. Males, Black people, and those with RCa had the worst OS.

**Conclusions:**

Using clinical, nutritional, and functional criteria, nearly one-quarter of the PDAC cases in our diverse, multi-institutional cohort had PCa and 62.5% had Ca or RCa at the time of diagnosis. The PCa estimate is higher than that reported in prior studies. We recommend these criteria be used to aid in CC classification, monitoring, and management of all incident PDAC cases. Findings also highlight the recommendation for continued emotional support, assistance in alleviating pain, and supportive care needs throughout the PDAC treatment journey.

## Introduction

Cancer-associated cachexia (CC) is a condition characterized by unintentional weight loss, loss of skeletal muscle mass (with or without fat loss), decreased appetite, fatigue, and other metabolic changes that limit therapeutic response, reduce quality of life (QoL), and decrease survival in 28–57% of cancer patients (1, 2). The prevalence of CC is highest in gastrointestinal malignancies and pancreatic ductal adenocarcinoma (PDAC) in particular, with up to 80% of PDAC patients affected during their disease (3), independent of resectability status (4–6). Proper early identification and treatment of CC has potential to improve QoL for patients newly-diagnosed with PDAC, but due to CC’s complex and heterogeneous pathophysiological and clinical characteristics, consensus is needed regarding its definition, the number of stages in its continuum, and classification criteria (7–22).

Historically, unintended weight loss greater than 5% was the only factor considered for CC classification, however recent literature suggests many factors be used (13). Based on landmark studies (12, 13, 17), Vigano and colleagues (18) developed a CC classification system using seven criteria to distinguish four CC stages [non-cachexia (NCa), pre-cachexia (PCa), cachexia (Ca), and refractory cachexia (RCa)]. In a subsequent study of patients with advanced cancer (59% with gastrointestinal cancers) (15), the Vigano classification (18) was modified to only include criteria readily accessible to clinicians encompassing four domains: 1) biochemistry (high C-reactive protein (CRP) or leukocytes, hypoalbuminemia, or anemia); 2) food intake (decreased); 3) weight loss over the past 6 months [moderate (≤5%) or significant (>5%)]; and 4) performance status (Eastern Cooperative Oncology Group (ECOG) Performance Status ≥ 3). A recent study conducted in Brazil (23) compared CC classification criteria from Vigano (15) with other groups (10, 16); among 1384 patients with advanced cancer (32% with gastrointestinal cancers), criteria by Vigano (15) were most effective in distinguishing between CC stages and predicting risk of 90-day mortality, though work is needed to better discriminate PCa from Ca and validate predictors of overall survival (OS) (15, 23).

Of studies that evaluated CC exclusively among patients with PDAC (24–31), the majority predated the Vigano publication (15), none used their (15) methods to classify CC stage, and nearly all classified cases as cachectic or non-cachectic (24–26, 28–31). Most studies included PDAC cases with any tumor stage (26, 28, 30, 31), while others focused only resectable disease (24, 25) or restricted to locally advanced or metastatic disease (27, 29). Additionally, the majority of studies of PDAC-associated cachexia did not evaluate patient-reported outcomes (PRO) as endpoints as recommended by the American Society of Clinical Oncology (22). Furthermore, practically all of these studies used a retrospective or cross-sectional design and did not collect measurements longitudinally to assess changes over time. Finally, except for one study from Taiwan (31), all studies of CC retrospectively analyzed homogeneous populations of Non-Hispanic White (NHW) or European PDAC cases. Only two studies reported inclusion of individuals who self-reported as Black people (26, 29). Based on the significantly higher PDAC incidence and mortality rates in African Americans (AA)/Black people compared to NHW and Hispanic Latinx (H/L) populations (32–44) and data from colorectal and esophageal cancers which showed Black people and H/L to have higher risks of presenting with CC compared to NHW (45), it seems prudent to prospectively evaluate PDAC-induced CC in racially and ethnically diverse populations.

The objectives of the current study were to 1) leverage the updated CC classification system by Vigano et al. (15) to determine the prevalence of CC stage at diagnosis among a diverse cohort of incident PDAC cases of all stages; 2) identify the prevalence of higher symptom burden, supportive care needs, and poor QoL, and factors predictive of these endpoints; and 3) examine the influence of individual factors on OS, controlling for tumor stage and resectability.

## Materials and methods

### Study population

This study included patients from 15 institutions (academic cancer centers and community hospitals) participating in the Florida Pancreas Collaborative (FPC), a state-wide population-based prospective cohort study and biobank conducted between May 2018 and September 2021 (46). Participants were eligible if they: a) were 18 years old and above, b) self-reported as African American or Black people, NHW, or H/L [the populations having the greatest PDAC burden in Florida and nationally (20)], c) were willing to complete the study questionnaire and donate biospecimens at the time of standard-of-care (SOC) procedures, and d) had a strong suspicion of PDAC and were treatment-naïve. The diagnosis was confirmed by pathologic review of tissue obtained through routine diagnostic procedures. The study was approved by the Advarra Institutional Review Board, and all patients provided informed consent to participate.

### Data collection

As described in a whitepaper from the FPC (46), data collection occurred at baseline/enrollment and at 6- and 12-months post-baseline via health screens, questionnaires, and case report forms. The 3-page health screen comprises the abridged version of the Patient-Generated Subjective Global Assessment (aPG-SGA), a revised version of the Edmonton Symptom Assessment System (ESAS-r), and the Canadian Problem Checklist (**Supplementary Figures 1A-C**). The aPG-SGA (47) is a four-part questionnaire that gathers self-reported information as part of “boxes” pertaining to: 1) height, weight, and weight change over the past two weeks, 2) food intake over the past month, 3) presence of symptoms affecting food intake over the past two weeks, and 4) activities and function over the past month. ESAS-r assesses the prevalence and severity of symptoms (e.g., pain, tiredness/fatigue) on a scale of 0 (“no symptoms”) to 10 (“worst symptoms”) (48). The Canadian Problem Checklist (CPC) is based on supportive care needs of cancer patients and includes domains of emotional, informational, practical, spiritual, social, family, and physical concerns (49, 50). Information on cigarette smoking over a lifetime and the past 30 days was also ascertained in the health screen.

Participants also completed a self-administered web- or teleform-based baseline questionnaire and abbreviated versions at follow-up time-points. The baseline questionnaire gathered demographic, socioeconomic, epidemiologic, and clinical variables. We also assessed self-reported grip strength via questionnaire since using a dynamometer was cost-prohibitive for our multi-center study (51). Two questions were asked: “Have you found it challenging to grip objects with your entire hand (such as when giving a hand-shake or when holding on to a stairwell or carrying a handle of a bucket)?” and “Have you found it challenging to grip objects with your fingers (such as a piece of paper or a coin)?” Responses included not at all, a little, quite a bit, and very much. Validated questionnaires were used to assess mental health, lifetime stress, nutrition, and QoL (52–54). Performance status was assessed at the clinic visit by treating providers using ECOG guidelines (55). Data was obtained from the electronic medical record (EMR) on presenting symptoms, anthropometric measures, comorbidities (56), primary tumor location (head, body, tail, other), imaging studies, surgical and pathology details, chemotherapy regimen, and lab values. Treatment and follow-up data from the Florida Cancer Data System (57) was also obtained. Vital status was verified using the National Death Index, Social Security Death Index, obituaries, autopsy reports, and death certificates.

### Laboratory values

Most participants had routine blood analyses performed as part of SOC, and had data on pre-treatment laboratory values considered in the Vigano classification (15) (CRP, albumin (Alb), hemoglobin (HgB), and white blood cell (WBC) count). Additionally, based on data supporting serum cancer antigen 19–9, the Glasgow Prognostic Score (GPS) (objective scoring based on concentrations of CRP and albumin), neutrophil to lymphocyte ratio (NLR), platelet count, and bilirubin as having prognostic value for patients with PDAC and other cancer types (58–65), these markers were also considered using previously-defined cut-offs (66–70). Since CRP was not routinely ordered as part of SOC for all participants, serum levels of CRP were evaluated as part of a multiplexed array research panel (**Supplementary Methods**) with other SOC markers and novel biomarkers of interest that are not a focus of Vigano (15) using archived serum from a subset of cases. Since biomarker concentrations may be influenced by antibiotics, steroids, or chemotherapy (27, 67, 71), neo- or adjuvant therapy and medication details were considered.

### Cancer cachexia stage

Participants were classified into four CC stages (NCa, PCa, Ca, and RCa) at baseline using criteria in **Table 1**: abnormal biochemistry (A), decreased food intake (B), minimal weight loss (C), significant weight loss (D), and decreased activities and functioning (E-F). Due to small numbers of cases with complete data in certain categories, a dichotomous CC status variable (cachectic vs. non-cachectic) was created based on criteria from Fearon et al. (13) wherein cachectic patients reported weight loss >5% over the past 6 months or had a BMI <20 and any weight loss >2% over the past 6 months and non-cachectic cases did not meet either threshold. Quality of life (QOL) was assessed using the European Organization for Research and Treatment of Cancer (EORTC) QLQ-C30 and QLQ-PAN-26 instruments. The QLQ-C30 comprises 30 questions based on QoL, function, and symptoms, and is intended to determine general health-related QoL (HRQoL) specific to cancer patients. With the exception of two items, scoring involves a 4-point Likert scale where 1= “Not at all”, 2= “A little”, 3= “Quite a bit”, and 4= “Very much”. Items 29 (overall health rating) and 30 (overall QoL) are scored on a 7-point ranking ranging from 1= “very poor” to 7= “excellent”. Scales were linearly transformed to a score from 0 to 100 (72) with 100 depicting the best health quality. The EORTC QLQ-PAN26 was validated in PDAC (73–75) and contains 26 items on self-reported symptoms and issues specific to PDAC such as abdominal discomfort and back pain. Higher scores signify poorer QoL for all scales except for the satisfaction scale for which higher scores denote better QoL.

**Table 1:** Criteria used for cancer cachexia classification^^^ in the Florida pancreas collaborative pancreatic ductal adenocarcinoma cohort.

Precachectic (PCa)	
A. Biochemistry^‡^	CRP>10 mg/L or Alb<3.2mg/dL or WBC count >11,000 or HgB <120g/L (men), <110 g/L (women)
B. Food intake	Decreased food intake (PG-SGA box 2, >1)
C. Minimal weight loss	≤ 5% WL over 6 months
Stage criteria	A+B or A+C or B+C or A+B+C
**Intervention**	**Monitor and/or refer to rehabilitation programs for preventive intervention.**
Cachectic (Ca)	
A. Biochemistry	CRP>10 mg/L or Alb<3.2mg/dL or WBC count >11,000 or HgB <120g/L (men), 110 g/L (women)
B. Food intake	Decreased food intake (PG-SGA box 2, >1)
D. Significant weight loss	> 5% WL over 6 months
E. Decreased activities/functioning	PG-SGA box 4, ≤2
Stage criteria	A+D+E or B+D+E or A+B+D+E
**Intervention**	**Multimodal management according to symptoms and patient wishes (prioritize reversible contributory factors)**
Refractory cachectic (RCa)	
A. Biochemistry	CRP>10 mg/L or Alb<3.2mg/dL or WBC count >11,000 or HgB <120g/L (men), <110 g/L (women)
B. Food intake	Decreased food intake (PG-SGA box 2, >1)
D. Significant weight loss	> 5% WL over 6 months
F. Decreased activities/functioning	*PG-SGA box 4, >2
Stage criteria	A+D+F or B+D+F or A+B+D+F or Alb<2.5mg/dL+D or Alb<2.5mg/dL+E or Alb<2.5mg/dL+D+F
**Intervention**	**Symptom palliation, psychosocial and nutritional support**
Noncachectic (NCa)	
	Does not meet any of the criteria above

Alb, albumin; CRP, C-reactive protein; HgB, hemoglobin; PG-SGA, Patient-generated Subjective Global Assessment.

WBC, white blood cell; WL, weight loss.

*PG-SGA score for box 4 >2 corresponds to ECOG performance score >3.

^Based on criteria from Vigano et al. (15) with modifications including: Omission of hand grip percentile in item E as a dynamometer is not always available in clinics. Patients with weight loss >5% with no other symptoms were placed in the cachectic category.

‡Variables used to differentiate a more severe cachectic stage from a less severe stage are highlighted in gray.

### Statistical analysis

Descriptive statistics were used to summarize baseline characteristics of the study population. For continuous variables, differences between groups were tested by ANOVA with means ± standard deviations (SD) followed by the Tukey Honest Significant Differences *post-hoc* test. For non-normally distributed variables, non-parametric Kruskal Wallis tests were performed and followed by Mann Whitney U-tests. Chi-square tests or McNemar’s chi square tests were used to determine differences in proportions for categorical variables. Symptoms were also categorized into groups based on the nature of the symptoms. Physical symptoms included six domains (pain, fatigue, drowsiness, nausea, loss of appetite, and shortness of breath). Psychological symptoms included two domains (anxiety and depression). The mean of all symptoms represented the total symptom subscore. Individual symptom scores and subscores obtained from ESAS-r were dichotomized into 0–3 (none to mild) or 4–10 (moderate to severe) (76, 77). The frequency of “none to mild” or “moderate to severe” physical, psychological, and total symptom subscore categories were compared across groups using chi-squared tests.

To identify factors predictive of moderate to severe symptom burden, supportive care needs, HRQoL, and/or CC status, multivariate logistic regression models were used. Pearson correlation tests were used to evaluate the linear relationship between laboratory values and other characteristics. The influence of individual factors and CC status on QoL was investigated with multivariate generalized linear regression. Due to possible collinearity of contributing factors, we performed preliminary global data exploration using multiple correspondence analysis (MCA) (78). MCA is an analog to principal component analysis for categorical variables and was carried out using the R package FactoMiner. A sum of cosine^2^ of >0.3 (a measure of how well each variable is represented by the first 2 dimensions) was used as a cutoff for modeling.

To evaluate associations with OS, we conducted Kaplan-Meier analysis and multivariate Cox proportional hazards (PH) regression, and calculated hazard ratios and 95% confidence intervals (CIs). OS was calculated from the date of diagnosis to the date of death from any cause or last follow-up. Covariates considered in analyses included age at diagnosis, race and ethnicity, gender, BMI, age-adjusted Charlson Comorbidity Index (ACCI), tumor stage, smoking history (never, past, or current), laboratory values, and treatment received (20, 21, 26, 28, 29, 79–82). Effect modification was evaluated by conducting stratified analysis by biological sex and racial and ethnic group. Analyses were performed using R 4.3.1.

## Results

### Demographic, socioeconomic, epidemiologic, and clinical characteristics of the cohort

A total of 318 PDAC cases (161 female, 157 male) were recruited to the FPC study. Select characteristics of the cohort overall and by race and ethnicity are in **Table 2**. The average age at diagnosis was slightly younger in AA (67.2 years) and H/L (66.0 years) compared to NHW (70.5 years), with 25.5% of H/L and 14.3% of AA diagnosed at age 55 or younger compared to 4.2% of NHW (P<0.001 for H/L and P=0.015 for AA pairwise comparisons). A higher proportion of females was observed among AA and H/L racial and ethnic groups compared to NHW, at 62.9%, 66%, and 45.8%, respectively, though significance was only observed for H/L versus NHW pairwise comparisons (P=0.011). NHW were more likely to be married and report a higher annual income than AA and H/L. No significant differences were observed between racial and ethnic groups in education level or insurance status. A higher proportion of never smokers were observed amongst H/L (72.3%) compared to NHW (39.4%), P<0.001. Differences were also observed between racial and ethnic groups for several anthropometric variables. Namely, BMI was slightly higher in AA (28.2 kg/m^2^) compared to H/L (24.6 kg/m^2^) and NHW (26.7 kg/m^2^), with approximately 69% of AA, 60% of NHW, and 42% of H/L reported to be overweight or obese. Waist to hip ratio and waist circumference were significantly lower in H/L compared to NHW, with P=0.0161, P=0.04, respectively (ANOVA and Tukey’s HSD).

**Table 2:** Select characteristics of the FPC pancreatic ductal adenocarcinoma cohort at enrollment, by race and ethnicity (n=318).

	Categories	All (N=318)	NHW (n=236)	AA (n=35)	H/L (n=47)	P-value (global)^¥^
**Age at diagnosis (mean, median (SD))**		69.4, 71 (9.8)	70.5, 72 (8.7)	67.2, 68 (10.4)	66.0, 68.5 (13.3)	0.094
**Age at diagnosis, n (%)**	≤55 years	27 (8.5)	10 (4.2)	5 (14.3)	12 (25.5)	**<0.001**
	>55 years	291 (91.5)	226 (95.7)	30 (85.7)	35 (74.5)	
**Sex, n (%)**	Female	161 (50.6)	108 (45.8)	22 (62.9)	31 (66.0)	**<0.001**
	Male	157 (49.4)	128 (54.2)	13 (37.1)	16 (34.0)	
**Education, n (%)**	Post-graduation	57 (17.9)	35 (14.8)	2 (5.7)	5 (10.6)	0.801
	Up to college	92 (28.9)	61 (25.8)	7 (20.0)	6 (12.8)	
	Up to high school	42 (13.2)	59 (25.0)	8 (22.9)	8 (17.0)	
	Missing	124 (39.0)	81 (34.3)	18 (51.4)	28 (59.6)	
**Marital Status, n (%)**	Married	53 (16.7)	113 (47.9)	9 (25.7)	15 (31.9)	**<0.001**
	Not married	137 (43.1)	39 (16.5)	8 (22.9)	8 (17.0)	
	Missing	128 (40)	84 (35.6)	18 (51.4)	24 (51.1)	
**Insurance Status, n (%)**	Private	68 (21.4)	53 (22.5)	6 (17.1)	9 (19.1)	0.411
	Medicaid	11 (3.5)	9 (3.8)	2 (5.7)	0 (0.0)	
	Medicare	107 (33.6)	88 (37.2)	8 (22.8)	11 (23.4)	
	Uninsured	2 (0.6)	1 (0.4)	0 (0.0)	1 (2.1)	
	Missing	132 (41.5)	87 (36.9)	19 (54.3)	26 (55.3)	
**Annual Income, n (%)**	$100K and above	34 (10.7)	33 (14.0)	1 (2.9)	0 (0.0)	0.057
	$40K-<$100K	63 (19.8)	51 (21.6)	5 (14.3)	7 (14.9)	
	Below $40K	55 (17.2)	41 (17.4)	9 (25.7)	5 (10.6)	
	Missing	130 (40.9)	82 (34.7)	18 (51.4)	30 (63.8)	
	Prefer not to say	38 (11.9)	31 (13.1)	2 (5.7)	5 (10.6)	
**Smoking status, n %**	Never	146 (45.9)	93 (39.4)	19 (54.3)	34 (72.3)	**0.002**
	Former	136 (42.78)	113 (47.9)	13 (37.1)	10 (21.3)	
	Current	31 (9.75)	25 (10.6)	3 (8.6)	3 (6.38)	
**Body mass index (kg/m^2^), n%**	Underweight <18.5	27 (8.7)	16 (7.0)	3 (8.6)	8 (17.8)	**0.049**
	Normal 18.5–24.9	102 (33.0)	76 (33.2)	8 (22.9)	18 (40.0)	
	Overweight/obese >25	180 (58.3)	137 (59.8)	24 (68.6)	19 (42.2)	
**Jaundice, n (%)**	Yes	99 (31.13)	70 (29.7)	9 (25.7)	20 (42.6)	0.225
	No	173 (54.4)	122 (51.7)	26 (74.3)	25 (53.2)	
**Weight loss >5%, n (%)**	Yes	193 (62.5)	141 (62.1)	22 (62.9)	30 (63.8)	0.54
	No	116 (37.5)	86 (37.9)	13 (37.1)	17 (36.2)	
**Cachexia Stage ^, n (%)**	Non-cachectic	40 (12.9)	34 (14.9)	3 (8.57)	3 (6.38)	0.111
	Pre-cachectic	76 (24.6)	52 (22.9)	10 (28.6)	14 (29.8)	
	Cachectic	167 (54.1)	127 (56.0)	18 (51.4)	22 (46.8)	
	Refractory cachectic	26 (8.41)	14 (6.2)	4 (11.4)	8 (17.02)	
Cachexia Stage *, n (%)	Non-cachectic	116 (37.5)	86 (37.9)	13 (37.1)	17 (36.2)	0.975
	Cachectic	193 (62.5)	141 (62.1)	22 (62.9)	30 (63.8)	
**ACCI, n (%)**	<=2	90 (28.3)	59 (25)	12 (34.3)	19 (40.4)	0.168
	2–5	138 (43.4)	130 (55.1)	5 (14.3)	3 (6.38)	
	>=6	45 (14.2)	37 (15.9)	5 (14.3)	3 (6.38)	
**Diabetes, n (%)**	Yes	99 (31.1)	73 (30.9)	9 (25.7)	17 (36.2)	0.227
	No	150 (47.1)	103 (43.6)	25 (71.4)	22 (46.8)	
	Missing	69 (21.7)	60 (25.4)	1 (2.8)	8 (17.0)	
**ECOG functional score, n (%)**	0	131 (41.2)	99 (41.9)	12 (34.3)	20 (42.6)	0.059
	1	146 (45.9)	109 (46.2)	21 (60.0)	16 (34.0)	
	2	19 (6.0)	15 (6.4)	2 (5.7)	2 (4.3)	
	3 or 4	19 (6.0)	2 (0.8)	0 (0)	3 (6.4)	
	Missing	17 (5.3)	11 (4.7)	0 (0)	6 (12.8)	
**Tumor location, n (%)**	Body	11 (3.5)	7 (3.0)	1 (2.9)	3 (6.4)	**0.043**
	Diffuse	53 (16.7)	40 (16.9)	7 (20.0)	6 (12.8)	
	Head	62 (19.5)	46 (19.5)	2 (5.7)	14 (29.8)	
	No information	152 (47.8)	114 (48.3)	18 (51.4)	20 (42.6)	
	Other	22 (6.9)	13 (5.5)	6 (17.1)	3 (6.4)	
	Tail	18 (5.7)	16 (6.8)	1 (2.9)	1 (2.1)	
**Stage, n (%)**	1	56 (17.6)	44 (18.6)	3 (8.6)	9 (19.1)	0.473
	2	78 (24.5)	59 (25.0)	6 (17.1)	13 (12.8)	
	3	38 (11.9)	26 (11.0)	5 (14.3)	7 (14.9)	
	4	84 (26.4)	61 (25.8)	13 (37.1)	10 (21.3)	
	NA	62 (19.4)	46 (26.3)	8 (22.9)	8 (31.9)	
**Grade, n (%)**	1	5 (1.6)	3 (1.3)	0 (0)	2 (4.3)	0.640
	2	77 (24.2)	58 (24.6)	7 (20.0)	12 (25.5)	
	3	52 (16.4)	37 (15.7)	6 (17.1)	9 (19.1)	
	NA	184 (57.9)	138 (58.5)	22 (62.9)	24 (51.1)	
**Treatment regimen, n (%)**	Surgery Only	98 (30.8)	77 (32.5)	10 (28.6)	11 (23.4)	0.870
	Non-surgical Only	84 (26.4)	58 (24.6)	14 (40.0)	12 (25.5)	
	Multi-Modal	111 (34.9)	82 (34.7)	9 (25.7)	20 (42.6)	
	Missing/Other	24 (7.5)	19 (8.1)	2 (5.7)	3 (6.4)	
**Surgical resection, n (%)**		209 (67.6)	159 (67.4)	19 (54.3)	31 (66.0)	NA
**Chemotherapy, n (%)**		108 (35.0)	72 (30.5)	15 (42.9)	21 (44.7)	
**Survival time in months,** **Median (95% CI)**		14.6 (13.5,16.6)	14.2 (12.4,16.7)	12.6 (10.3,16.3)	22.5 (16.6,NA^‡^)	**0.018**
**Vital Status n (%)**	Alive	121 (38.1)	91 (38.5)	9 (25.7)	21 (46.7)	NA
	Dead	180 (56.6)	132 (55.9)	25 (71.4)	23 (48.9)	
	Unknown	17 (5.3)	13 (5.6)	1 (2.9)	3 (6.4)	

Numbers and percentages may not add up to 100 because of missing data. P-values of <0.05 are in **bold**.

AA, African American; ACCI, Age-adjusted Charlson Comorbidity index; CI, confidence interval; ECOG, Eastern Cooperative Oncology Group; FPC, Florida Pancreas Collaborative; H/L, Hispanic/Latinx; NA, Not applicable; NHW, non-hispanic White.

**^¥^** p-values indicate the global p-values for ANOVA, Kaplan-Meier estimation or chi-square tests where appropriate.^ Cachexia staging according to Vigano et al (2017) modified as follows: hand grip strength was not used to assess cachexia stage and any patient with >5% weight loss in the absence of any other factor was assigned to the cachectic category. Cachexia stage could not be assessed for 9 individuals due to missing weight values.

*Cachexia staging using weight loss only to categorize patients as cachectic or non-cachectic using criteria by Fearon et al. (17).

^‡^Upper CI could not be calculated due to too few events at later time points.

Although no statistically significant differences were observed between racial and ethnic groups regarding their personal history of diabetes or the number of comorbidities, a slightly lower proportion of AA participants (34.3%) reported to be fully active/able to carry out pre-disease performance without restriction (ECOG performance status=0) at baseline compared to NHW (41.9%) and H/L (42.6%). Weight loss >5% over the prior 6 months was the most common presenting symptom across all racial and ethnic groups, affecting 62.5% of the cohort. Of cases with known tumor stage documented (n=256; 80.5%), 42.1% had stage I/II disease. When stratified by race and ethnicity, the highest proportion of stage III/IV cases was among AA (51.4%) followed by NHW (36.8%) and H/L (36.2%). Nearly 35% of cases underwent multimodal treatment (defined as receipt of curative-intent surgery and chemotherapy), with receipt of multi-modal treatment highest among H/L (42.6%) and lowest among AA (25.7%). Survival time was significantly longer among H/L (median 22.5 months, 95% CI: 16.6-unestimatable) compared to NHW (14.2 months 95% CI: 12.4–16.7) and AA (12.6 months 95% CI: 10.3–16.3) (P=0.016, log-rank test).

### Cancer cachexia stage and status

CC status was determined for 309 (97.1%) of the 318 cases with available data. Using criteria from Vigano et al. (15), the prevalence of NCa, PCa, Ca, and RCa at enrollment was 12.9%, 24.6%, 54.1%, and 8.4%, respectively (**Table 2**; **Supplementary Figure 2**). Thus, approximately 87% of participants were deemed to have pre-cachexia, cachexia, or refractory disease at the time of diagnosis when using the five criteria (15). When using weight loss only to categorize participants as in (13), only 62.5% of individuals were classified as cachectic. More severe stages of CC were observed among males (NCa 10.5%; PCa 17.6%; Ca 62.1%; RCa 9.8%) than females (NCa 15.4%; PCa 31.4%; Ca 46.2%; RCa 7.5%). H/L had the highest proportion of cases with refractory cachexia at diagnosis (17%) followed by AA (11.4%) and NHW (6.2%), though these differences were not statistically significant (P=0.111, univariate chi square test) (**Table 2**). No associations between baseline BMI and CC stage at baseline were detected (data not shown).

### Biochemical variables

The prevalence of the criterion variables used to categorize CC stage is in **Table 3**; **Supplementary Figure 3**. CRP was available in the EMR for only 7% of study participants. We therefore used blood from a subset of participants [n=202; 14.6% AA, 66% NHW, 19.4% H/L)] who donated it for serum processing (46) and evaluated CRP levels using a multiplex panel (**Supplementary Methods**). As expected, the prevalence of abnormal CRP (>10 mg/L) was higher in the RCa cohort (53.8%) compared to 0% of NCa, 25% of PCa, and 16.7% of Ca cases, with statistically significant differences (P<0.001) in CRP levels between CC stages. WBC levels >11,000/mL increased with CC severity, and were observed in 11.8%, 13.17%, and 34.62% of PCa, Ca, or RCa cases, respectively (P=0.0008). Similar to the higher prevalence of abnormal CRP levels among cases classified as PCa compared to Ca, abnormal albumin levels (<3.2 mg/dl) were more common in cases with PCa (11.84%) versus Ca (6.59%). Using a <3.2 mg/dl threshold to identify patients with PCa or Ca appeared to be more sensitive than the lower threshold of albumin (<2.5 mg/dl) used by Vigano et al. (15). Abnormal HgB levels were more prevalent in males than females across the CC continuum, affecting nearly two-thirds of patients classified as having refractory disease. The prevalence of abnormal HgB levels was highest among RCa cases followed by PCa and Ca (P=0.0418 for females, P=0.0007 for males). Laboratory values not considered by Vigano et al. (15) that were significantly different between CC stages using a Cochrane-Armitage trend test included NLR (P=0.0365), GPS (P=0.0059) and bilirubin (P<0.001).

**Table 3:** Prevalence of criteria considered for cancer cachexia stage classification at baseline in the FPC PDAC cohort.

		NCa (n=40)		PCa (n=76)		Ca (n=167)		RCa (n=26)	
		24 F, 16 M		(49 F, 27 M)		(72 F, 95 M)		(11 F, 15 M)	
CRP>10 mg/L^a^	NA	39	97.50%	70	92.11%	155	92.81%	24	92.31%
	No	1	2.50%	4	5.26%	5	2.99%	0	0.00%
	Yes	0	0.00%	2	2.63%	7	4.19%	2	7.69%
CRP>10 mg/L^b^	NA	12	30.00%	23	30.26%	64	38.32%	8	30.77%
	No	28	70.00%	34	44.74%	75	44.91%	4	15.38%
	Yes	0	0.00%	19	25.00%	28	16.77%	14	53.85%
**WBC> 11,000 mL**	NA	5	12.50%	1	1.32%	3	1.80%	1	3.85%
	No	35	87.50%	66	86.84%	142	85.03%	16	61.54%
	Yes	0	0.00%	9	11.84%	22	13.17%	9	34.62%
**Albumin <3.2 mg/dL**	NA	6	15.00%	3	3.95%	5	2.99%	1	3.85%
	No	34	85.00%	64	84.21%	151	90.42%	18	69.23%
	Yes	0	0.00%	9	11.84%	11	6.59%	7	26.92%
**Albumin <2.5 mg/dL**	NA	6	15.00%	3	3.95%	5	2.99%	1	3.85%
	No	34	85.00%	72	94.74%	162	97.01%	19	73.08%
	Yes	0	0.00%	1	1.32%	0	0.00%	6	23.08%
**HgB <11 for females**	NA	2	8.33%	1	2.04%	0	0.00%	0	0.00%
	No	22	91.67%	36	73.47%	58	80.6%	7	63.64%
	Yes	0	0.00%	12	24.49%	14	19.4%	4	36.36%
**HgB <12 for males**	NA	1	6.25%	0	0.00%	3	3.16%	1	6.67%
	No	15	93.75%	18	66.67%	63	66.32%	4	26.67%
	Yes	0	0.00%	9	33.33%	29	30.52%	10	66.67%
CA 19–9 >37 U/ml^c^	NA	7	17.50%	21	27.63%	29	17.37%	9	34.62%
	No	9	22.50%	12	15.80%	25	14.97%	5	19.23%
	Yes	24	60.00%	43	56.58%	113	67.66%	12	46.15%
GPS =2^d^	NA	15	37.50%	24	31.58%	68	40.72%	8	30.77%
	No	25	62.50%	49	64.47%	92	55.09%	13	50.00%
	Yes	0	0.00%	3	3.95%	7	4.19%	5	19.23%
NLR >=5^e^	NA	17	42.50%	25	32.89%	41	24.55%	5	19.23%
	No	19	47.50%	40	52.63%	89	77.84%	12	46.15%
	Yes	4	10.00%	11	14.47%	37	22.16%	9	34.62%
Platelet Ct>300 x 10^3^/uL^f^	NA	4	10.00%	1	1.32%	1	0.60%	1	3.85%
	No	32	80.00%	52	68.42%	126	75.45%	17	65.38%
	Yes	4	10.00%	23	30.26%	40	23.95%	8	30.77%
Bilirubin> 1.1 mg/dL^g^	NA	5	12.50%	4	5.26%	5	2.99%	2	7.69%
	No	32	80.00%	52	68.42%	71	42.51%	11	42.31%
	Yes	3	7.50%	20	26.31%	91	54.49%	13	50.00%
**Weight loss >5%**	NA	0	0.00%	0	0.00%	0	0.00%	0	0.00%
	No	40	100.00%	76	100.00%	0	0.00%	0	0.00%
	Yes	0	0.00%	0	0.00%	167	100.00%	26	100.00%
**aPG-SGA box2 score >1**	No	40	100.00%	68	89.47%	139	83.23%	16	61.54%
**(food intake)**	Yes	0	0.00%	8	10.53%	28	16.77%	10	38.46%
**aPG-SGA box4 score >2 (activity score)**	No Yes	40 0	100.00% 0.00%	73 3	96.05% 3.95%	167 0	100.00% 0.00%	4 22	15.38% 91.67%

Criteria in bold font were significantly different (P<0.05) between cachexia stages. Percentages may not add up to 100 due to rounding. CRP, C reactive protein; GPS, Glasgow Prognostic Score; WBC, white blood cells; HgB, hemoglobin; PC, platelet count; aPG-SGA, abridged version of the Patient-Generated Subjective Global Assessment; N/L ratio, neutrophil to lymphocyte ratio; F, female; M, male; NCa, non-cachectic; PCa, pre-cachectic; Ca, Cachectic; RCa, Refractory cachexia; NA, Results are not available in the electronic medical record (EMR) and/or cannot be estimated; PG-SGA, Patient-Generated Subjective Global Assessment.

a Values obtained from the EMR using standard of care testing.

b Values obtained from a research-grade laboratory assay.

c CA19–9 cut-off value from Poruk et al, Cur Mol Med 2013. PMID: 23331006.

d Patients with an elevated CRP level (>1.0 mg/dl) and hypoalbuminemia (<3.5 g/dl) were assigned a GPS score of 2 which is considered high (Scores of 0 or 1 are in the low category). Per PMID 32606164 by Kurosaki et al. (In Vivo, 2020), those with only one of these abnormalities were assigned a score of 1, and those with neither a score of 0.

e Cut-off value for NLR is from PMID 30349646 from Ogata et al. and PMID: 32606164 from Kurosaki et al.

f Platelet count (PC) cut off value from Dominguez et al. (World J Surg, 2008), PMID 18224462.

g Bilirubin cut off value from Zhu et al. (Int J Biol Markers, 2021), PMID 34374580.

An exploratory evaluation of biomarker levels by race and ethnicity and CC status revealed AA patients were significantly more likely to present with normal CA19–9 levels than other racial and ethnic groups regardless of CC status (**Supplementary Table 1**), in line with other studies (83). NLR was also more likely to be in the low/normal category among AA, especially among those with CC, with P=0.051. Finally, bilirubin levels were more likely to be abnormal among AA patients with cachexia than patients in the other racial and ethnic groups.

### Nutritional and functional variables

Nearly two-thirds of the 309 cases (n=193; 83 women,110 men) reported loss of >5% of their body weight in the 6 months prior to their diagnosis and were classified as having Ca or RCa (**Tables 2**, **3**). Decreased food intake (aPG-SGA box 2 score >1) was observed in approximately 15% of cases (n=46) and was most prevalent among those with RCa. Reduced activities and functioning (aPG-SGA box 4 score >2) was reported in 8.1% of cases overall and in nearly 92% of the RCa cases (**Table 3**). No statistically significant differences were observed by race and ethnicity for weight loss, food intake, or activities/functioning. Of 178 participants who completed questions about hand-grip strength, 148 (83.1%) responded “not at all” to challenges related to hand or finger grip strength, 28 individuals reported having “a little” or “quite a bit” of challenges, and only 2 individuals (with RCa) reported challenges as “very much.” Concordance between self-reported grip strength and dynamometer measurements is unclear.

### Symptom burden, supportive care needs, and HRQoL

Symptom burden, which includes symptom type, prevalence, and severity, is summarized overall and by sex and race and ethnicity in **Supplementary Table 2**, **Supplementary Figure 4**. At baseline, physical and psychological symptom sub-scores were moderate to severe in 43 (14.1%) and 82 (26.9%) of all participants, respectively. The total symptom score was moderate to severe in 19% of cases (n=58). The most commonly-reported physical symptoms across all racial and ethnic groups included fatigue (n=145, 45.5%), loss of appetite (n=108, 35.4%), pain (n=85, 27.9%), and drowsiness (n=68, 22.3%). Of psychological symptoms, anxiety (n=104, n=34.1%) and depression (n=67, 22.0%) were the most frequently reported. While the mean physical subscore was significantly higher among cases with stage III/IV versus stage I/II disease (2.07 versus 1.61, P=0.0354), the median psychological subscore did not differ by stage (P=0.7211) (data not shown). Physical and psychological subscores tended to be higher among females compared to males, though they were not statistically different. More severe anxiety was reported at a higher frequency among females compared to males (P=0.011) and at a lower frequency among AA compared to H/L and NHW (P=0.0346). The prevalence of moderate to severe nausea was significantly higher among H/L and AA participants compared to NHW. Importantly, evaluation of changes in symptom burden over the first 6 months post-diagnosis showed that while mean “tiredness/fatigue” scores nominally increased, “anxiety” and overall psychological scores decreased (**Supplementary Table 3**).

The problems and supportive care needs reported by participants at baseline are in **Supplementary Table 4**, **Supplementary Figure 5**. Of 314 participants who completed the Canadian Problem Checklist, emotional concerns ranked first by 177 cases (56.4%) who expressed fears, worries, frustration, and anger. Physical changes involving sleep and weight were the next most frequently cited concerns affecting 46.5% of patients followed by informational concerns (n=130, 41.4%) related to understanding their illness and/or treatment options. No statistically significant differences in concerns were evident when comparing participants by sex or race and ethnicity. Assessment of supportive care needs over the first 6-month time-period revealed significant changes in all major categories (Emotional, Physical, Spiritual, Practical, Informational and Social) when compared to the “None” category (**Supplementary Table 5**), with significant trends downwards. In contrast, concerns related to concentration/memory, frustration/anger, and worries about changes in appearance increased over time.

**Figure 1:**
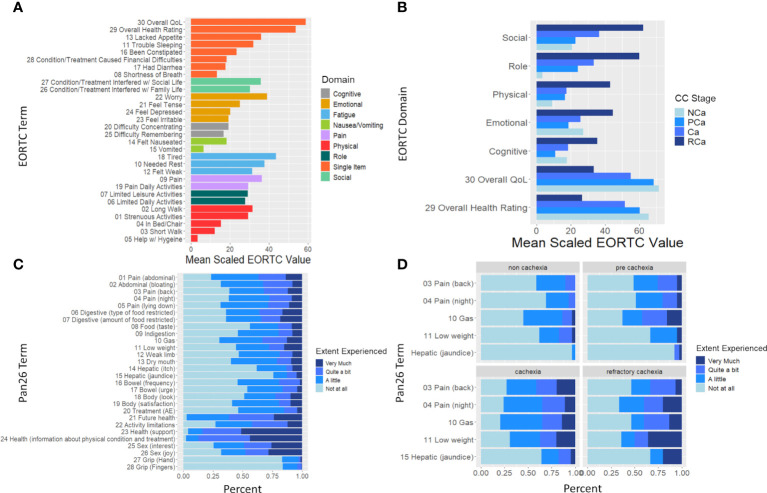
EORTC and PAN-26 heath-related quality of life (HRQoL) variables differ significantly based on cachexia stage at baseline. **(A)** Bar graph depicting mean of the EORTC QLQ30 scaled to 100 and colored by either EORTC domain (if a multi-item domain) or single-item (if item is part of a single-item domain). **(B)** Bar graph depicting mean scaled EORTC QLQ 30 domain and the overall QoL and health rating stratified by cachexia stage. Brackets indicate p<0.05 between RCa and all other categories (ANOVA). **(C)** Frequency of scores (“not at all”, “a little”, “quite a bit”, very much”) at baseline for the PAN-26 HRQoL questionnaire. **(D)** Frequency of scores of the 5 PAN-26 variables which were found to be significantly different by cachexia stage at baseline.

HRQoL was evaluated by 180 of 318 PDAC cases. “Tiredness”, “worry”, and “pain” were the main symptoms experienced using the QLQ-30 (**Figure 1A**). Stratification by cachexia status further demonstrated that all functional domains and overall HRQoL significantly worsen as cachexia stage progresses (**Figure 1B**). The distribution of responses to QLQ-PAN26 items plus two items concerning grip strength is shown overall and by cachexia stage in **Figures 1C, D**. The extent of pain, restrictions in the type and amount of food, and weight loss negatively affected HRQoL, especially among AA and H/L. H/L also reported fewer/less severe side effects from treatment than NHW and AA. Evaluation of responses to the QLQ-PAN26 over time indicates that the “most improved” symptoms are “food taste”, “treatment side effects”, “limited in doing planned activities” and “information from healthcare providers,” each with 56.14% of participants reporting improvements (**Supplementary Table 6**). Conversely, 42.11% and 47.37% of patients reported a deterioration in HRQoL from “back pain” and “pain at night” respectively. Joy from intimacy, abdominal bloating, and digestive issues worsened for a significant proportion of participants.

### Predictors of higher symptom burden, supportive care needs, HRQoL, and CC status

MCA (78) of symptom burden, supportive care needs, and HRQoL variables indicated that ~36% of variability was explained by dimensions 1 and 2. ESAS-r categories of pain, appetite, and tiredness/fatigue explained most of the variability along dimension 1, PAN-26 symptom “Gas” explained the majority of variability along dimension 2, and ESAS-r scores for anxiety and depression were explanatory for both dimensions (**Supplementary Figure 6**). Interestingly and perhaps because physical symptoms may arise from varying clinical sequelae, clustering was only observed in the ESAS-r psychological domain. Binomial regression indicated male sex was negatively associated with an ESAS-r “tiredness” score of >=4 (P=0.03, OR=0.44;CI:0.21,0.90) and an “anxiety” score of >=4 (OR=0.39; 95%CI:0.15,0.95, P=0.045), and positively associated with high bilirubin (P=0.001, OR=2.18; 95%CI:1.06,4.59). Cachexia status was also associated with fatigue, although CIs were wide. Interestingly, patients with a GPS score of >1 were *less* likely to report “Gas” as a burden (OR=0.158; 95%CI:0.02,0.90, P=0.046). When CC status was modeled via ordinal regression using clinicopathological and demographic predictor variables, we found a significant association between CC status and GPS score >1 (P=0.030; OR=3.67; 95%CI:0.87,12.22). Male sex and late-stage disease also approached significance (P=0.13 and 0.065 respectively). Variance inflation factors were <2 (data not shown).

### Associations between key covariates and OS

**Figure 2:**
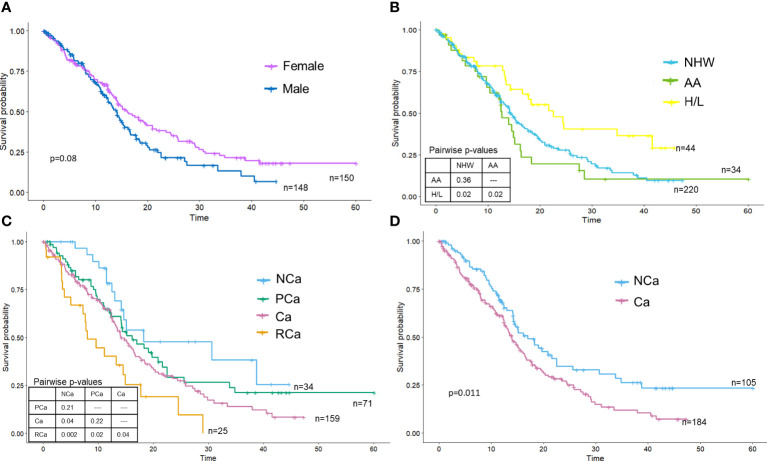
Overall survival is significantly different by race and ethnicity and cachexia stage. Kaplan-Meier survival curves were plotted and stratified by **(A)** sex, **(B)** race and ethnicity, **(C)** cachexia stage (Vigano criteria) or **(D)** cachexia stage (Fearon criteria).

The median OS of the PDAC cohort was 14.6 months (95% CI: 13.5–16.6 months), with surgical patients having better OS than patients not receiving surgery. Females had slightly longer OS than males, though differences did not reach statistical significance (log-rank P=0.08) (**Figure 2A**). Kaplan Meier curves portrayed that race and ethnicity influenced OS, with H/L having significantly improved survival (HR: 0.57, 95% CI: 0.36–0.89) compared to NHW and AA (**Figure 2B**). AA had the poorest survival (HR:1.22, 95% 0.79–1.87), but survival time was not statistically different from NHW (P=0.37). Kaplan Meier curves for the four CC stages (15) are in **Figure 2C** and showed a significant difference (log-rank P=0.0034) between the stages. As expected, those with RCa had the poorest survival (HR: 3.24; 95% CI: 1.63–6.44, log-rank P=0.003) and those with NCa experienced the best survival. No significant differences in survival were observed between the PCa and Ca groups; Ca (HR: 1.87 CI: 1.07–3.28) but not PCa (HR: 1.50 CI: 0.82–2.75) was significantly associated with mortality in univariate analysis. Cachexia status [e.g., cachectic vs non-cachectic using the sole criteria of weight loss over 6 months (13)] also showed significant differences (log-rank test P=0.011) (**Figure 2D**), with cachectic participants having poorer survival on univariate analysis (HR: 1.51, 95% CI: 1.10–2.09). Although a formal analysis of survival for biological sex stratified by race and ethnicity was not performed due to small sample sizes for minorities, we did note an increase in median survival for females for both H/L (female median=34.8 months vs male median=16.6 months) and NHW (female median=16.1 months vs male median=13.5 months) but not AA (female median=12.4 months vs male median=14.9 months) racial and ethnic groups.

**Figure 3:**
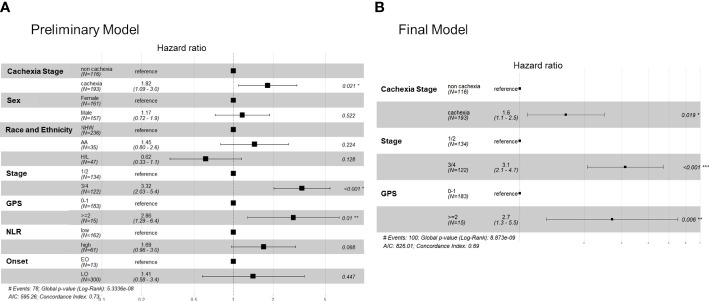
Cox proportional hazards models showing the association between key covariates and overall survival in the FPC cohort. Forest plots for the **(A)** preliminary (full) cox survival model (without the treatment variable) and **(B)** the finalized model.

Finally, based on findings of univariate analysis we performed PH modeling of OS using cachexia status, biological sex, race and ethnicity (NHW as reference), stage (III/IV vs I/II), GPS (>=2 or <2), NLR (high versus low), and age (early versus late-onset) as predictor variables. Our preliminary model indicated that only cachexia status (HR: 1.82, 95% CI: 1.09–3.0, P=0.021), stage (HR: 2.53, 95% CI: 1.39–4.61, P=0.0002) and GPS (HR:2.48, 95% CI: 1.03–5.96, P=0.01) were significantly associated with survival (**Figure 3A**). When including treatment status (multi-modal versus single therapy), only stage and GPS predicted survival, and upon further interrogation as expected single-therapy treatment status (e.g., palliative/systemic chemotherapy) was highly associated with stage III/IV unresectable disease (P<0.001). In a final more parsimonious PH model, cachectic status, late stage, and a high GPS were all associated with decreased survival times; NLR was not significant in the final model (**Figure 3B**).

## Discussion

Early recognition and management of CC among individuals diagnosed with PDAC stem from the mission to improve treatment tolerability, optimize QoL, and prolong OS. To date, studies of PDAC-associated CC have been limited by: the varying definitions of CC used; the lack of racially and ethnically diverse populations studied; the retrospective or cross-sectional designs, and different endpoints evaluated, with a paucity of studies including QoL as a primary endpoint despite evidence-based guidance (22). To develop a consensus classification system for PDAC cachexia, clinical validation must occur via prospective studies with well-defined cohorts and clearly stated outcomes. The current multi-institutional study of PDAC is the first in the US we know of to publish on data obtained longitudinally from a diverse cohort as part of routine assessments to classify CC stage along its continuum and identify factors predictive of higher symptom burden, supportive care needs, QoL, and OS.

When using clinical, nutritional, and functional CC criteria recommended by Vigano and colleagues (15), nearly 25% of PDAC cases were classified as having pre-cachexia, 54.1% had cachexia, and 8.4% had refractory disease. In line with existing data (20, 26, 45, 84–89), a higher prevalence and severity of CC and slightly worse survival was observed in males. The estimate of pre-cachexia overall (25%) is higher than that reported in a study of unresectable PDAC by Wesseltoft-Rao and colleagues (27) which estimated the prevalence of pre-cachexia [using the classification by Fearon et al. (13)] to be 15% (27). The prevalence of cachexia was 60% among patients in that study (27). Using the sole criterion of weight loss over the prior 6 months to categorize participants as cachectic or non-cachectic as in Fearon et al. (13), 62.5% of individuals in our study were classified as cachectic. This estimate aligns with studies of PDAC-associated cachexia which categorized participants as cachectic or non-cachectic using weight loss only. For example, in a retrospective evaluation of 977 PDAC patients seen over an 8-year time-period at Kaiser Permanente (26), 63% of patients were diagnosed with cachexia (defined as weight loss >5% over the 6 months prior to diagnosis), regardless of stage. In a cohort of 227 patients with resectable PDAC (24), the prevalence of cachexia at the time of resection was 40% (defined as unintended weight loss greater than 10% of the pre-illness stable body weight). Had weight loss >5% been considered (24), the prevalence would have been 60.8%. Although weight loss assessment was evaluated in our study using self-report, short- and long-term patient-reported weight history has demonstrated validity and reproducibility compared to measured body weight (90–92). Since weight loss may serve as a marker of systemic disease, our findings and that of others suggest weight should be assessed at baseline and actively monitored throughout the patients’ cancer journey to assess early weight loss. Notably, there was a high correlation (r=0.98) between self-reported weight and objectively-measured weight at baseline among FPC participants.

Consistent with a retrospective analysis of PDAC (26), gastroesophageal and colorectal cancers (45), and lung cancers (93), there was a higher prevalence of CC at diagnosis in AA compared to NHW. However, to our knowledge, this is the first report of PDAC cases to show that H/L had a trend towards a higher prevalence of CC (and RCa in particular) yet longer survival compared to NHW and AA. Studies from other cancer types have also reported a higher prevalence of CC at diagnosis among H/L compared to NHW (45, 93, 94). Social determinants of health such as access to private insurance, education level, and income do not appear to influence racial and ethnic differences in CC prevalence or survival in our cohort. Furthermore, significant differences in tumor stage and treatment regimen received were not observed between the racial and ethnic groups. The greater cachexia prevalence in minority groups may therefore depend on social support, environmental, biological, or other constructs and mechanisms not yet evaluated. Indeed, chronic stress weathering has been highlighted as a potential factor in minority health disparities (95). The state of Florida is one of the lowest performing states (ranks number 44) in terms of health care access and affordability (96). Indeed, the proportion of FPC PDAC cases who reported on household income in the lowest bracket mimics that of cases state-wide using the Florida Cancer Data System. Taken together, the prevalence of the CC continuum (and PCa in particular) is very high at the time of PDAC diagnosis in our diverse and representative patient population, especially when using classification criteria from Vigano and colleagues (15).

In the FPC cohort, we found a significant difference in survival between the four stages of CC (15), with patients with refractory disease experiencing the worst survival and non-cachectic individuals experiencing the best survival. However, no significant differences in survival were observed between the PCa and Ca groups. Our findings align with studies of other cancers (10, 15, 18, 27, 97) which also did not find a significant difference in survival between pre-cachectic and cachectic patients. Explanations for these findings have been suggested (15), and include heterogeneity in the pre-cachectic group since it may comprise patients at high risk for cachexia as well as those already in an early cachectic phase. It is also possible that imprecision lies in subjective recall of weight changes, food intake, or activities, and that monitoring changes in these criteria over time using more objective measures may be required to discriminate further between these two stages. Nevertheless, in our study and that of Vigano et al. (15), non-cachectic cases lived longer and had a different prevalence of biochemical, nutritional, and functional variables than pre-cachectic individuals, suggesting that PCa status identified patients with forthcoming cachexia (27). We therefore contend that: classification of PCa is clinically relevant, this group is a prime target for intervention, and opportunities exist to better educate clinicians about how to identify and manage cachexia (98–100).

We found statistically significant differences between CC stages in the prevalence of WBC counts and CRP, albumin, and HgB levels, with the most abnormal values in patients with RCa. However, abnormal CRP, albumin, and HgB levels were more frequently reported among patients with PCa versus those with Ca. Of note, CRP was not routinely available in the EMR of FPC PDAC cohort participants; CRP merits evaluation at baseline and throughout treatment. Furthermore, because significant differences were identified between CC stages for NLR, GPS, and bilirubin, these markers should be considered in future longitudinal studies.

Nutritional and functional variables have not been considered in most existing studies of PDAC-associated cachexia. Although patients may purposely alter their diets and naturally lose weight after a cancer diagnosis (101, 102), decreased food intake was observed in approximately 15% of cases and was most prevalent among those with RCa, as in other studies (15). Although the aPG-SGA tool does not assess actual food intake, self-report of a “less than usual intake” has been shown to estimate reduced intake <1500 kcal/day (103, 104) and is far less time-consuming for a patient to complete compared to a daily dietary record. A reduced score for activities and functioning was reported in 8.1% of the subjects overall. Taken together, uniform, easy to use, cost-effective, accurate and objective methods to improve assessment of food intake and functioning could be valuable for cachexia monitoring and treatment.

Evaluating symptom burden and supportive care needs is crucial to mitigate physical and psychological comorbidities early and improve coping and treatment compliance (105). However, to our knowledge, few studies have been published on the routine collection of PRO among patients with PDAC-associated cachexia throughout the course of diagnosis and treatment. Based on self-report, at baseline physical and psychological symptoms were moderate to severe in 14.1% and 26.9% of all cohort participants, respectively. Consistent with published data (19), the most frequently reported physical symptoms included fatigue, loss of appetite, and pain, and the most common psychological symptoms were anxiety and depression. In our study, symptom intensity tended to be higher among females compared to males, in line with prior research (77). We also found that anxiety was reported at a significantly lower frequency among AA compared to H/L and NHW. The most commonly-reported problems and supportive care needs reported at baseline were emotional concerns related to fears and worries and physical changes involving sleep and weight. Fears and worries were also the highest-ranking problem amongst cancer patients in a study by Cuthbert and colleagues (105). Trends upwards over time were observed for physical concerns related to concentration/memory and emotions related to frustration/anger. Taken together, despite implementation of routine collection of PROs at cancer centers, these data underscore a call to action for early and ongoing assessment and interventions to address physical and psychological symptoms and supportive care needs and potentially other parameters including financial toxicity in real time to improve care delivery and QoL for patients with PDAC. Furthermore, given that only ~30% of PDAC patients receive early palliative care referral at end-of-life, this study provides support for the adoption of early supportive care models regardless of disease stage (106).

HRQoL evaluated by the EORTC QLQ-30/PAN26 has been found to be understandable and relevant among clinicians and patients with locally advanced or metastatic PDAC (107). These instruments revealed “tiredness”, “worry”, and “pain” as the main symptoms experienced by the FPC cohort, with symptoms significantly worsening with more advanced CC stages. The extent of pain, restrictions in the type and amount of food, and weight loss negatively affected HRQoL, especially among AA and H/L. Indeed, studies suggest race and ethnicity may influence the way functional limitations and well-being are perceived and reported (108, 109). Evaluation of responses to the more specific QLQ-PAN26 over time showcased 42.11% and 47.37% of patients reported worsening of “back pain” and “pain at night” respectively. Significant worsening in pain, abdominal discomfort, and sexual dissatisfaction was also reported in a study of PDAC patients 5 years post pancreatoduodedenctomy (110). Abdominal and back pain are among the most common symptoms of PDAC (and often serve as an indicator of unresectability and/or recurrence) (19, 111–114), with a recent report suggesting that approximately 75% of PDAC patients experience pain, and >50% of them have cachexia (115) The pathophysiology of pain in PDAC is complex and multifactorial and may be attributed to tumor invasion, pancreatic enzyme insufficiency, obstruction of ducts, and/or nerve involvement due to entrapment or infiltration of the dense network of nerves around the pancreas (111, 114, 116). Strategies for pain management include pancreatic enzyme therapy (for exocrine insufficiency) and range from acetaminophen and non-steroidal anti-inflammatory drugs and opioids to radiation therapy, neurolysis, intrathecal drug delivery, and integrative approaches such as nutraceuticals, acupuncture, and exercise physiotherapy (111, 114, 116), with some studies suggesting higher efficacy of earlier implementation of invasive treatment in reducing pain, preventing deterioration in QoL, and lengthening overall survival (116, 117). Our findings emphasize the critical need for timely and continued supportive care in the short and long term for all patients with PDAC to help decrease ongoing cancer-related pain according to its severity and origin. Additionally, great merit exists in developing and testing additional interventions to improve pain in partnership with patients and in examining PROs before and after administration of interventions.

Despite the study strengths, there are limitations and opportunities we are addressing with further research. Although data support the use of dual-energy X-ray absorptiometry (DXA) and computed tomography (CT) to evaluate lean body mass (118) and the loss of muscle mass and strength that occurs with age (e.g., sarcopenia) and in the presence of PDAC (119), Vigano et al. (15) did not incorporate these metrics into their CC criteria, likely due to the lack of implementation research to showcase incorporation of body composition analysis in real-time clinically. The FPC cohort does have longitudinally collected CT images and will be reporting on measures of muscle mass and quality and adiposity at baseline and changes over time in the future. Another opportunity for CC research is the discovery and validation of novel blood-based biomarkers that could aid in diagnosing earlier stages of CC, identifying patients at increased risk for treatment-related toxicities, and monitoring therapeutic effects. We are finalizing analysis of longitudinally collected serum samples from FPC cohort participants to evaluate cytokines, adipokines, chemokines, and other analytes. It is our intent to incorporate CT body composition data and novel serum biomarker data with data described in this study to assess their added value in improving prediction of CC stage (and pre-cachexia in particular) QoL, and survival.

In summary, our study demonstrated that classification based on data available in routine clinical practice can be leveraged to identify and characterize the presence and severity of cachexia and predict several endpoints in patients with PDAC. When compared to the NCa stage, PCa, Ca, and RCa were indeed associated with significant differences in clinical, nutritional, and functional measures and in outcomes across sexes and racial and ethnic groups. Pre-cachexia is difficult to identify but here, using the Vigano criteria (15), we identify 76/309 patients (24.6%) as pre-cachectic. This represents a significant number of people who could be targeted for early intervention. However, we fully acknowledge future longitudinal studies and objective measures may be needed to more accurately distinguish between pre-cachexia and cachexia and enable more timely and personalized interventions for patients with PDAC. Our findings support guidance from key stakeholders in the PDAC and cachexia community (19, 21, 22, 114, 120) which recommend a multidisciplinary approach inclusive of symptom management, nutrition and pharmacologic intervention, physical therapy, psychosocial support, resistance training, and targeted therapeutic agents. Based on our findings, management of pain should be addressed as early as possible to improve QoL. We also suggest that multidisciplinary care, especially referrals to a registered dietitian and supportive care team, occur pre-emptively for all patients with PDAC, regardless of weight loss, so they can be assessed for their appetite, nutritional intake, exocrine insufficiency, and hormonal and micronutrient deficiencies. Studies by members of our team also support the feasibility, acceptability, usability, and preliminary efficacy of remote nutrition and exercise monitoring interventions in cancer survivorship (121–127).

## Data availability statement

The raw data supporting the conclusions of this article will be made available by the authors, without undue reservation.

## Ethics statement

The studies involving humans were approved by Advarra Institutional Review Board. The studies were conducted in accordance with the local legislation and institutional requirements. The participants provided their written informed consent to participate in this study. Written informed consent was obtained from the individual(s) for the publication of any potentially identifiable images or data included in this article.

## Author contributions

JP: Conceptualization, Data curation, Formal analysis, Funding acquisition, Methodology, Writing – original draft, Writing – review & editing, Investigation, Resources, Supervision. MP: Data curation, Formal analysis, Methodology, Writing – original draft, Writing – review & editing, Conceptualization, Visualization. D-TC: Formal analysis, Methodology, Writing – review & editing. TB: Data curation, Project administration, Writing – review & editing. BP: Writing – review & editing. CG: Writing – review & editing. KD: Data curation, Project administration, Writing – review & editing, Supervision. MG: Data curation, Project administration, Writing – review & editing. SV: Data curation, Project administration, Writing – review & editing, Formal Analysis. TBi: Writing – review & editing. EC: Writing – review & editing, Data curation, Project administration. SC: Writing – review & editing. MG-D: Data curation, Project administration, Writing – review & editing. BG: Writing – review & editing. AG: Writing – review & editing. CGr: Writing – review & editing. SH: Writing – review & editing. KJ: Writing – review & editing. BK: Data curation, Writing – review & editing. VV: Writing – review & editing. JG: Writing – review & editing. AM: Data curation, Writing – review & editing. QM: Writing – review & editing. LM-U: Data curation, Writing – review & editing. SM: Writing – review & editing. NP: Writing – review & editing. SR: Writing – review & editing. GR: Writing – review & editing. AS: Writing – review & editing. LS: Writing – review & editing. PS: Writing – review & editing. KT: Writing – review & editing, Data curation. AT: Writing – review & editing. JT: Writing – review & editing. DT: Writing – review & editing, Data curation. KT: Writing – review & editing. SVa: Writing – review & editing. CW: Writing – review & editing. WD: Writing – review & editing. VV: Writing – review & editing. AK: Writing – review & editing. AL: Writing – review & editing. KM: Writing – review & editing. MM-V: Writing – review & editing. KH: Writing – review & editing. JA: Writing – review & editing. MB: Writing – review & editing. JT: Funding acquisition, Writing – review & editing, Conceptualization. NM: Writing – review & editing. JPi: Writing – review & editing. PH: Writing – review & editing. MM: Writing – review & editing. JF: Writing – review & editing. SJ: Conceptualization, Methodology, Writing – original draft, Writing – review & editing. DJ: Conceptualization, Data curation, Writing – original draft, Writing – review & editing. AJ: Conceptualization, Methodology, Writing – original draft, Writing – review & editing.
