# Supplementary material for: Leveraging real-world data to predict cancer cachexia stage, quality of life, and survival in a racially and ethnically diverse multi-institutional cohort of treatment-naïve patients with pancreatic ductal adenocarcinoma
Source: Front Oncol. 2024 Jul 23;14:1362244. doi: 10.3389/fonc.2024.1362244 (PMC11300308; doi:10.3389/fonc.2024.1362244)
Supplement: Supplementary file 8 [file Table_2.docx]

**Supplementary Table 2. ESASr Symptom Scores at Baseline for FPC PDAC Cohort Participants Overall and by Sex and Race and Ethnicity**

|  | **Overall (n=305)** | | | | **Males (n=152)** | | | | **Females (n=153)** | | | | **Non-Hispanic White (n=223)** | | | | **African American (n=35)** | | | | **Hispanic/Latino (n=47)** | | | |  |
| --- | --- | --- | --- | --- | --- | --- | --- | --- | --- | --- | --- | --- | --- | --- | --- | --- | --- | --- | --- | --- | --- | --- | --- | --- | --- |
|  |  |  |  |  |  |  |  |  |  |  |  |  |  |  |  |  |  |  |  |  |  |  |  |  |  |
| **Individual symptom scores** | None-mild (0-3) n % | | Moderate- severe  (4-10) n % | | None-mild (0-3) n % | | Moderate- severe  (4-10) n % | | None- mild (0-3) n % | | Moderate- severe  (4-10) n % | | None to mild (0-3) n % | | Moderate- severe  (4-10) n % | | None- mild (0-3) n % | | Moderate- severe  (4-10) n % | | None- mild (0-3) n % | | Moderate-  severe  (4-10) n % | |  |
|  |  |  |  |  |  |  |  |  |  |  |  |  |  |  |  |  |  |  |  |  |  |  |  |  |  |
| Pain | 229 | 75.1 | 85 | 27.9 | 117 | 77.0 | 35 | 23.0 | 103 | 67.3 | 50 | 32.7 | 167 | 74.9 | 56 | 25.1 | 24 | 68.6 | 11 | 31.4 | 29 | 61.7 | 18 | 38.3 |  |
| Tiredness | 159 | 52.1 | 145 | 47.5 | 80 | 52.6 | 71 | 46.7 | 79 | 51.6 | 74 | 48.4 | 119 | 53.4 | 104 | 46.7 | 17 | 48.6 | 17 | 48.6 | 23 | 48.9 | 24 | 51.1 |  |
| Drowsiness | 237 | 77.7 | 68 | 22.3 | 117 | 77.0 | 35 | 23.0 | 120 | 78.4 | 33 | 21.6 | 171 | 76.7 | 52 | 23.3 | 27 | 77.1 | 8 | 22.9 | 39 | 83.0 | 8 | 17.0 |  |
| **Nausea*** | 274 | 89.9 | 31 | 10.2 | 139 | 91.5 | 13 | 8.6 | 135 | 88.2 | 18 | 11.8 | **206** | **92.4** | **17** | **7.6** | **30** | **85.7** | **5** | **14.3** | **38** | **80.9** | **9** | **19.2** |  |
| Loss of  appetite | 197 | 64.6 | 108 | 35.4 | 103 | 67.8 | 49 | 32.2 | 94 | 61.4 | 59 | 38.6 | 141 | 63.2 | 82 | 36.8 | 26 | 74.3 | 9 | 25.7 | 30 | 63.8 | 17 | 36.2 |  |
| Shortness of  breath | 274 | 89.8 | 30 | 9.8 | 136 | 89.5 | 15 | 9.9 | 138 | 90.2 | 15 | 9.8 | 204 | 91.5 | 19 | 8.5 | 27 | 79.4 | 7 | 20.6 | 43 | 91.5 | 4 | 8.5 |  |
| Depression | 238 | 78.0 | 67 | 22.0 | 118 | 77.6 | 34 | 22.4 | 120 | 78.4 | 33 | 21.6 | 171 | 76.7 | 52 | 23.3 | 32 | 91.4 | 3 | 8.6 | 35 | 74.5 | 12 | 25.5 |  |
| Anxiety | 201 | 65.9 | 104 | 34.1 | 108 | 71.1 | 44 | 29.0 | 93 | 60.8 | 60 | 39.2 | 140 | 62.8 | 83 | 37.2 | 27 | 77.1 | 7 | 20.0 | 33 | 70.2 | 14 | 29.8 |  |
| Well-being | 220 | 72.1 | 85 | 27.9 | 78 | 51.3 | 74 | 48.7 | 79 | 51.6 | 74 | 48.4 | 167 | 74.9 | 56 | 25.1 | 24 | 68.6 | 11 | 31.4 | 29 | 61.7 | 18 | 38.3 |  |
| Other | 276 | 90.5 | 21 | 6.9 | 139 | 91.5 | 8 | 5.3 | 137 | 89.5 | 13 | 8.5 | 202 | 90.6 | 14 | 6.3 | 31 | 88.6 | 3 | 8.6 | 43 | 91.5 | 4 | 8.5 |  |
| **Subscores** |  |  |  |  |  |  |  |  |  |  |  |  |  |  |  |  |  |  |  |  |  |  |  |  |  |
| Physical  symptoms | 262 | 85.9 | 43 | 14.1 | 131 | 86.2 | 21 | 13.8 | 131 | 85.6 | 22 | 14.4 | 192 | 86.1 | 31 | 13.9 | 32 | 91.4 | 3 | 8.6 | 38 | 80.9 | 9 | 19.2 |  |
| Psychological  symptoms | 223 | 73.1 | 82 | 26.9 | 110 | 72.4 | 42 | 27.6 | 113 | 73.9 | 40 | 26.1 | 157 | 70.4 | 66 | 29.6 | 31 | 88.6 | 4 | 11.4 | 35 | 74.5 | 12 | 25.5 |  |
|  |  |  |  |  |  |  |  |  |  |  |  |  |  |  |  |  |  |  |  |  |  |  |  |  |  |
| Total  symptoms | 241 | 79.0 | 58 | 19.0 | 119 | 78.3 | 30 | 19.7 | 122 | 79.7 | 28 | 18.3 | 173 | 77.6 | 45 | 20.2 | 32 | 91.4 | 3 | 8.6 | 36 | 76.6 | 10 | 21.3 |  |

*** Nausea was significantly different between RE groups based on a univariate chi-square test (symptoms = ‘none’ versus symptoms = ‘yes’)**
