# Supplementary material for: Leveraging real-world data to predict cancer cachexia stage, quality of life, and survival in a racially and ethnically diverse multi-institutional cohort of treatment-naïve patients with pancreatic ductal adenocarcinoma
Source: Front Oncol. 2024 Jul 23;14:1362244. doi: 10.3389/fonc.2024.1362244 (PMC11300308; doi:10.3389/fonc.2024.1362244)
Supplement: Supplementary file 9 [file Table_3.docx]

**Supplementary Table 3. ESASr Symptom Prevalence and Means and Changes Over Time for FPC PDAC Cohort Participants**

|  | **Prevalence**  **N=305 (Baseline)**  **N=102 (Follow-up)** | | | | **Mean**  **Symptom Score** | | | | **Significant Difference in Mean Scores** | **Improvement**  **<-1** | | **Stable**  **>-1 to <1** | | **Deterioration >1** | |
| --- | --- | --- | --- | --- | --- | --- | --- | --- | --- | --- | --- | --- | --- | --- | --- |
| **Individual symptom scores** | **At Baseline N %** | | **At Follow-up (6mo)**  **N %** | | **At Baseline Mean (SD)** | | **At Follow-up (6mo)**  **Mean (SD)** | | **P** | **N %** | | **N %** | | **N %** | |
| None | 30 | 9.84 | 12 | 11.8 | 0 | 0 | 0 | 0 | N/S | 9 | 8.8 | 90 | 88.2 | 2 | 2.0 |
| Pain | 85 | 27.9 | 26 | 25.5 | 1.79 | 2.54 | 1.31 | 2.05 | **0.01103** | 16 | 15.7 | 49 | 48.0 | 37 | 36.3 |
| Tiredness | 145 | 47.5 | 67 | 65.7 | 3.00 | 2.92 | 3.25 | 2.91 | **0.004341** | 42 | 41.2 | 32 | 31.4 | 28 | 27.5 |
| Drowsiness | 68 | 22.3 | 34 | 33.3 | 1.44 | 2.37 | 1.63 | 2.46 | 0.07433 | 32 | 31.4 | 42 | 41.2 | 26 | 25.5 |
| Nausea | 31 | 10.2 | 11 | 10.8 | 0.69 | 1.80 | 0.54 | 1.37 | 0.7365 | 14 | 13.7 | 74 | 72.5 | 14 | 13.7 |
| Loss of  appetite | 108 | 35.4 | 28 | 27.5 | 2.20 | 3.06 | 1.46 | 2.91 | 0.1272 | 23 | 22.5 | 45 | 44.1 | 34 | 33.3 |
| Shortness of  breath | 30 | 9.8 | 15 | 14.7 | 0.67 | 1.70 | 0.81 | 1.97 | 0.08323 | 18 | 17.6 | 76 | 74.5 | 7 | 7.9 |
| Depression | 67 | 22.0 | 25 | 24.5 | 1.42 | 2.48 | 1.32 | 2.43 | 0.1294 | 23 | 22.5 | 52 | 51.0 | 27 | 6.9 |
| Anxiety | 104 | 34.1 | 36 | 35.3 | 2.43 | 2.94 | 1.85 | 2.51 | **0.01865** | 21 | 20.6 | 39 | 38.2 | 42 | 41.2 |
| Well-being | 85 | 27.9 | 56 | 54.9 | 3.30 | 2.70 | 2.87 | 2.56 | 0.4883 | 43 | 42.2 | 16 | 15.7 | 43 | 26.5 |
| Other | 21 | 6.9 | 25 | 24.5 | 0.52 | 1.72 | 1.21 | 2.40 | 0.9953 | 15 | 14.7 | 52 | 51.0 | 10 | 9.8 |
| **Subscores** |  |  |  |  |  |  |  |  |  |  |  |  |  |  |  |
| Physical  symptoms | 43 | 14.1 | 21 | 20.6 | 1.63 | 1.59 | 1.52 | 1.50 | 0.7493 | 43 | 42.2 | 16 | 15.7 | 42 | 41.2 |
| Psychological  Symptoms | 82 | 26.9 | 27 | 26.5 | 1.92 | 2.38 | 1.53 | 2.23 | **0.01497** | 24 | 23.5 | 32 | 31.4 | 46 | 45.1 |

Note: Prevalence was calculated by the percentage of the cohort reporting at least one or more symptom at a value of >3.

P value was calculated using a paired Wilcoxon rank sum test.
