# Supplementary material for: Leveraging real-world data to predict cancer cachexia stage, quality of life, and survival in a racially and ethnically diverse multi-institutional cohort of treatment-naïve patients with pancreatic ductal adenocarcinoma
Source: Front Oncol. 2024 Jul 23;14:1362244. doi: 10.3389/fonc.2024.1362244 (PMC11300308; doi:10.3389/fonc.2024.1362244)
Supplement: Supplementary file 10 [file Table_4.docx]

**Supplementary Table 4**. Supportive care problems and concerns^ over the past week at baseline for FPC PDAC Cohort Participants Overall and by Sex and Race and Ethnicity

|  | **Overall**  **(n=314)** | | **Males**  **(n=157)** | | **Females**  **(n=157)** | | **Non-Hispanic White**  **(n=232)** | | **African American**  **(n=35)** | | **Hispanic/Latino**  **(n=47)** | |
| --- | --- | --- | --- | --- | --- | --- | --- | --- | --- | --- | --- | --- |
|  | N | % | N | % | N | % | N | % | N | % | N | % |
| **None** | 59 | 18.78 | 31 | 19.75 | 28 | 17.39 | 42 | 17.80 | 9 | 25.71 | 8 | 17.02 |
| **Emotional** | **177** | **56.37** | **84** | **53.50** | **92** | **58.60** | **131** | **56.47** | **16** | **45.71** | **29** | **61.70** |
| Fears/Worries | 136 | 20.06 | 66 | 42.04 | 70 | 44.59 | 103 | 44.40 | 11 | 31.43 | 22 | 46.81 |
| Sadness | 71 | 11.78 | 31 | 19.75 | 40 | 25.48 | 52 | 22.41 | 6 | 17.14 | 13 | 27.66 |
| Frustration/Anger | 63 | 5.10 | 30 | 19.11 | 33 | 21.02 | 51 | 21.98 | 7 | 20.00 | 5 | 10.64 |
| Changes in  appearance | 37 | 4.46 | 16 | 10.19 | 21 | 13.38 | 24 | 10.34 | 5 | 14.29 | 8 | 17.02 |
| Intimacy/Sexuality | 16 | 43.31 | 13 | 8.28 | 3 | 1.91 | 12 | 5.17 | 1 | 2.86 | 3 | 6.38 |
| Changes in who I am | 136 | 22.61 | 8 | 5.10 | 6 | 3.82 | 12 | 5.17 | 1 | 2.86 | 1 | 2.13 |
| **Spiritual** | **38** | **12.10** | **15** | **9.55** | **23** | **14.65** | **22** | **9.48** | **7** | **20.00** | **9** | **19.15** |
| Meaning/purpose of  life | 16 | 5.10 | 5 | 3.18 | 11 | 7.01 | 10 | 4.31 | 2 | 5.71 | 4 | 8.51 |
| Faith | 31 | 9.87 | 13 | 8.28 | 18 | 11.46 | 18 | 7.76 | 6 | 17.14 | 7 | 14.89 |
| **Practical** | **88** | **28.03** | **45** | **28.66** | **43** | **27.39** | **58** | **25.00** | **13** | **37.14** | **17** | **36.17** |
| Work/school | 27 | 8.60 | 13 | 8.28 | 14 | 8.92 | 18 | 7.76 | 1 | 2.86 | 8 | 17.02 |
| Finances | 56 | 17.83 | 27 | 17.20 | 29 | 18.47 | 33 | 14.22 | 10 | 28.57 | 13 | 27.66 |
| Getting to and from  appointments | 25 | 7.96 | 13 | 8.28 | 12 | 7.64 | 21 | 9.05 | 3 | 8.57 | 1 | 2.13 |
| Accommodation | 5 | 1.59 | 1 | 0.64 | 4 | 2.55 | 3 | 1.29 | 1 | 2.86 | 1 | 2.13 |
| Quitting smoking | 20 | 6.37 | 8 | 5.10 | 12 | 7.64 | 18 | 7.76 | 0 | 0.00 | 2 | 4.26 |
| **Social/Family** | **124** | **39.49** | **58** | **36.94** | **66** | **42.04** | **92** | **39.66** | **10** | **28.57** | **22** | **46.81** |
| Feeling like a burden | 51 | 16.24 | 24 | 15.29 | 27 | 17.20 | 41 | 17.67 | 2 | 5.71 | 8 | 17.02 |
| Worry about  family/friends | 97 | 30.89 | 44 | 28.03 | 53 | 33.76 | 74 | 31.90 | 8 | 22.86 | 15 | 31.91 |
| Feeling alone | 17 | 5.41 | 6 | 3.82 | 11 | 7.01 | 11 | 4.74 | 4 | 11.43 | 2 | 4.26 |
| **Informational** | **130** | **41.40** | **59** | **37.58** | **71** | **45.22** | **96** | **41.38** | **13** | **37.14** | **21** | **44.68** |
| Understanding my  illness and/or  treatment | 100 | 31.85 | 47 | 29.94 | 53 | 33.76 | 75 | 32.33 | 10 | 28.57 | 15 | 31.91 |
| Talking with the  health care team | 34 | 10.83 | 14 | 8.92 | 20 | 12.74 | 28 | 12.07 | 0 | 0.00 | 6 | 12.77 |
| Making treatment  decisions | 51 | 16.24 | 24 | 15.29 | 27 | 17.20 | 35 | 15.09 | 6 | 17.14 | 10 | 21.28 |
| Knowing about  available resources | 48 | 15.29 | 24 | 15.29 | 24 | 15.29 | 35 | 15.09 | 4 | 11.43 | 9 | 19.15 |
| Taking medications as  prescribed | 22 | 7.01 | 11 | 7.01 | 11 | 7.01 | 16 | 6.90 | 2 | 5.71 | 4 | 8.51 |
| **Physical** | **146** | **46.50** | **74** | **47.13** | **72** | **45.86** | **109** | **46.98** | **14** | **40.00** | **23** | **48.94** |
| Concentration/Memory | 53 | 16.88 | 26 | 16.56 | 27 | 17.20 | 43 | 18.53 | 6 | 17.14 | 4 | 8.51 |
| Sleep | 90 | 28.66 | 46 | 29.30 | 44 | 28.03 | 70 | 30.17 | 5 | 14.29 | 15 | 31.91 |
| Weight | 85 | 27.07 | 47 | 29.94 | 38 | 24.20 | 64 | 27.59 | 8 | 22.86 | 13 | 27.66 |

^Using the Canadian Problem Checklist. Note that participants can choose more than one supportive care need, so % does not equal 100.
