# Supplementary material for: Leveraging real-world data to predict cancer cachexia stage, quality of life, and survival in a racially and ethnically diverse multi-institutional cohort of treatment-naïve patients with pancreatic ductal adenocarcinoma
Source: Front Oncol. 2024 Jul 23;14:1362244. doi: 10.3389/fonc.2024.1362244 (PMC11300308; doi:10.3389/fonc.2024.1362244)
Supplement: Supplementary file 11 [file Table_5.docx]

**Supplementary Table 5. Supportive care problems and concerns^ of FPC PDAC cases over time**

|  | **Prevalence** | | | | **Significant difference in problems or concerns over time** |
| --- | --- | --- | --- | --- | --- |
|  | **At baseline**  **(n=314)** | | **At follow up time point 1* (n=251)** | | **P^#^** |
|  | N | % | N | % |  |
| **None** | 59 | 18.78 | 18 | 11.61 | N/A |
| **Emotional** | **177** | **56.37** | **56** | **36.13** | **< 0.001** |
| Fears/Worries | 136 | 20.06 | 35 | 22.58 | **< 0.001** |
| Sadness | 71 | 11.78 | 22 | 14.19 | **< 0.001** |
| Frustration/Anger | 63 | 5.10 | 25 | 16.13 | **< 0.001** |
| Changes in appearance | 37 | 4.46 | 21 | 13.55 | **0.01522** |
| Intimacy/Sexuality | 16 | 43.31 | 11 | 7.10 | 0.8638 |
| Changes in who I am | 136 | 22.61 | 10 | 6.45 | **< 0.001** |
| **Spiritual** | **38** | **12.10** | **4** | **2.58** | **0.01112** |
| Meaning/purpose of life | 16 | 5.10 | 3 | 1.94 | 0.8638 |
| Faith | 31 | 9.87 | 1 | 0.65 | 0.08648 |
| **Practical** | **88** | **28.03** | **17** | **10.96** | **< 0.001** |
| Work/school | 27 | 8.60 | 9 | 5.81 | 0.233 |
| Finances | 56 | 17.83 | 8 | 5.16 | **< 0.001** |
| Getting to/from appointment | 25 | 7.96 | 2 | 1.29 | 0.3602 |
| Accommodation | 5 | 1.59 | 1 | 0.65 | **0.01234** |
| Quitting smoking | 20 | 6.37 | 3 | 1.94 | 0.8711 |
| **Social/Family** | **124** | **39.49** | **45** | **29.03** | **< 0.001** |
| Feeling like a burden | 51 | 16.24 | 23 | 14.84 | **< 0.001** |
| Worry about family/friends | 97 | 30.89 | 33 | 21.29 | **< 0.001** |
| Feeling alone | 17 | 5.41 | 9 | 5.81 | 1.0 |
| **Informational** | **130** | **41.40** | **25** | **16.13** | **< 0.001** |
| Understanding my illness  and/or treatment | 100 | 31.85 | 13 | 8.39 | **< 0.001** |
| Talking with health care team | 34 | 10.83 | 5 | 3.23 | **0.03751** |
| Making treatment decisions | 51 | 16.24 | 7 | 4.52 | **< 0.001** |
| Knowing about resources | 48 | 15.29 | 10 | 6.45 | **< 0.001** |
| Taking medicine as prescribed | 22 | 7.01 | 4 | 2.58 | 0.6353 |
| **Physical** | **146** | **46.50** | **59** | **38.06** | **< 0.001** |
| Concentration/Memory | 53 | 16.88 | 31 | 20.00 | **< 0.001** |
| Sleep | 90 | 28.66 | 32 | 20.65 | **< 0.001** |
| Weight | 85 | 27.07 | 30 | 19.35 | **< 0.001** |

^Using the Canadian Problem Checklist.

*Median of 195 days between baseline and follow up timepoint 1.

^#^ P estimated using McNemar’s test for paired subjects for “None” versus the problem or concern listed in the row.

Note that participants can choose more than one supportive care need, so % does not equal 100.
