# Supplementary material for: Leveraging real-world data to predict cancer cachexia stage, quality of life, and survival in a racially and ethnically diverse multi-institutional cohort of treatment-naïve patients with pancreatic ductal adenocarcinoma
Source: Front Oncol. 2024 Jul 23;14:1362244. doi: 10.3389/fonc.2024.1362244 (PMC11300308; doi:10.3389/fonc.2024.1362244)
Supplement: Supplementary file 13 [file DataSheet_1.docx]

# Supplementary Figure Legends

# Supplementary Figure 1. The Florida Pancreas Collaborative ‘Health Screen’ Instrument. This instrument comprises (A) the abridged version of the Patient-Generated Subjective Global Assessment (aPG-SGA), (B) a revised version of the Edmonton Symptom Assessment System (ESAS-r), and (C) the Canadian Problem Checklist.

**Supplementary Figure 2. Cancer cachexia continuum prevalence A) overall, B) by sex, and C) by race and ethnicity.** Note that the percentages depicted in the bar graph for race/ethnicity are for the prevalence of precachexia, cachexia, and refractory cachexia, respectively.

**Supplementary Figure 3. Expression levels of biochemical markers, by cancer cachexia stage.** Boxplots overlaid with a jittered scatterplot of biochemical markers used to stage cachexia with the addition of the neutrophil to lymphocyte ratio (NLR). Values were log2 transformed prior to plotting.

**Supplementary Figure 4. Prevalence of moderate to severe symptom burden
at baseline (ESAS-r), overall and by sex and race and ethnicity.**

**Supplementary Figure 5. Supportive care problems and concerns over the past week at baseline for FPC PDAC cohort participants.**

**Supplementary Figure 6. ESAS scores for the “psychological” domain demonstrate clustering in a multiple correspondence analysis. (A-C)**: MCA plots of individuals colored by dichotomized ESAS scores of low (“lo”, <4) or high (“hi” >=4) for the psychological domain (mean scores, **A**), anxiety single item **(B)** or depression single item **(C)**. **(D)** MCA biplot of individuals (blue) and variables (red).
