# Supplementary material for: Leveraging real-world data to predict cancer cachexia stage, quality of life, and survival in a racially and ethnically diverse multi-institutional cohort of treatment-naïve patients with pancreatic ductal adenocarcinoma
Source: Front Oncol. 2024 Jul 23;14:1362244. doi: 10.3389/fonc.2024.1362244 (PMC11300308; doi:10.3389/fonc.2024.1362244)
Supplement: Supplementary file 14 [file DataSheet_2.docx]

**Supplementary methods**

**C-reactive protein (CRP) Values.** CRP values were derived from a multiplex ELISA utilizing Meso Scale Discovery’s (MSD) MULTI-ARRAY technology (Meso Scale Diagnostics, Rockville, MD) per manufacturer’s instructions. Samples were assayed in duplicate on 96-well plates and the electrochemiluminescence of bound secondary antibodies was measured on a MESO QuickPlex SQ120. Four parameter logistic curves and unknown concentration calculations were achieved using the DISCOVERY WORKBENCH 4.0 Analysis Software. Serum analyte concentrations were reported as pg/mL. Values higher or lower than the detection limit were reported as undetectable and excluded from the analysis.
